# A new species of sublittoral marine gastrotrich, *Lepidodasys ligni* sp. n. (Macrodasyida, Lepidodasyidae), from the Atlantic coast of Florida

**DOI:** 10.3897/zookeys.289.4764

**Published:** 2013-04-12

**Authors:** Rick Hochberg, Sarah Atherton, Vladimir Gross

**Affiliations:** 1 University of Massachusetts Lowell, One University Avenue, Lowell, MA 01854 USA, 01.978.934.2885

**Keywords:** Meiofauna, Florida, sublittoral, Capron Shoal, taxonomy, confocal

## Abstract

A new species of *Lepidodasys* is described from sublittoral sandy sediments off the Atlantic coast of Florida. *Lepidodasys ligni* **sp. n.** is a small species (≤ 450 µm) with a crossed-helical pattern of small, non-keeled, non-imbricated scales on the dorsal and lateral body surfaces, two columns of ventral, interciliary scales that form a herringbone pattern, and a series of anterior, lateral, dorsal and posterior adhesive tubes. Similar to *Lepidodasys castoroides* from the Faroe Islands, the new species possesses a caudal constriction that demarcates the posterior end containing the caudal organ. The frontal organ lies within the posterior constriction, which is heavily invested with somatic circular muscles. These muscles are also present throughout the trunk and represent a novel condition for species of *Lepidodasys*,which were previously considered to lack somatic circular muscles. Posterior of the caudal constriction is a large, barrel-shaped caudal organ that is wrapped in a series of interdigitating, spindle-shaped, incomplete circular muscle fibers. The caudal organ contains a sclerotized central canal, but the absence of distal cuticular endpieces distinguishes the new species from its morphologically similar congener, *Lepidodasys castoroides*.

## Introduction

Marine gastrotrichs are common members of the meiobenthos in intertidal and subtidal sandy sediments worldwide, and recent studies of various waters around the Caribbean – from the southern Atlantic waters of Florida to Panama - have revealed a wealth of previously undescribed species (Hochberg 2008, 2010; Hummon 2010; Hochberg and Atherton 2010, 2011; Atherton and Hochberg 2012; Kieneke et al. 2013a, b; Todaro & Leasi 2013). Species of *Lepidodasys* are exceptional among marine macrodasyidan gastrotrichs because of their pharyngeal architecture, muscular organization, unique spermatogenesis and the structure of their sculpted cuticle (see Ruppert 1978, 1991; Guidi et al. 2004). Indeed, these unique qualities are what prompted Hummon and Todaro (2010) to erect the monogeneric family, Lepidodasyidae. While over 400 species of marine gastrotrichs have been described worldwide (Hummon 2009), only eight species of *Lepidodasys* are described, which includes five species from European waters (Remane 1926, 1927; Balsamo et al. 1994; Clausen 2000, 2004), two species from Japanese waters (Lee and Chang 2011) and one species from the Caribbean (Hochberg and Atherton 2011).

While most species of *Lepidodasys* are identified based on the structure of their cuticle and the distribution and abundance of their adhesive tubes, two species are known to possess large reproductive organs that further differentiate them from the remaining members of the genus. *Lepidodasys castoroides* Clausen, 2004 possesses a large, barrel-shaped caudal organ with cuticular endpieces, and *Lepidodasys tsushmainensis* Lee & Chang, 2011 possesses a pyriform-shaped caudal organ without cuticular endpieces. During a recent sampling expedition to Capron Shoal, Florida, we discovered a previously undescribed species of *Lepidodasys* with a caudal organ reminiscent of the two aforementioned species. Herein, we describe this new species and provide detailed information on its caudal organ using f-actin staining and confocal laser scanning microscopy.

## Methods

In June 2012, approximately 18 liters of sediment were collected via anchor dredge from 9 m depth at Capron Shoal, Florida (27°26’52” N, 80°13’81” W). Meiofauna were extracted from the sediments using the anesthetization-decantation techinique with 7% MgCl_2_ and a 53 µm mesh. Specimens were sorted with a Leica EZ4 stereomicroscope, transferred to a glass slide, and viewed with a Zeiss A1 Axioscope equipped with DIC (differential interference contrast) and a Sony Handycam digital camera. Measurements of all specimens were performed with an ocular micrometer, and the size and positions of various organs are described in terms of percentage body units: anterior (U00) to posterior (U100) is 100 units.

For phalloidin staining of f-actin, we fixed two specimens in 4% paraformaldehyde in 0.1M phosphate buffer saline (PBS, pH 7.3) for 1 h, rinsed them in 0.1M PBS + 1% Triton X-100 (PBT) for 1 hr, and then stained them in Alexa Fluor 488 Phalloidin for 1 hr (following the manufacturer’s protocol, Invitrogen). Stained specimens were then briefly rinsed in PBS and mounted directly in Fluoromount G on glass microscope slides with coverslips.

Phalloidin-stained specimens were examined on a Zeiss LSM 510 confocal microscope system at the Smithsonian Marine Station (Fort Pierce, Florida). Zeiss Zen 2009 software (Carl Zeiss Microimaging, Thornwood, NY) was used to collect a series of 0.25-0.4 mm optical sections with maximum intensity projection along the z-axis. Confocal images were saved as TIF files and rendered into 3-D images using Volocity software (Perkin Elmer). Carnoy V 2.0 (© 2001 Peter Schols) was used to measure various organs in the digital images.

One stained specimen was removed from Fluoromount G after examination and prepared for museum archival with the following procedure: 1 hr rinse with PBS followed by 1% OsO_4_ in 0.1M PBS for 10 min (to increase contrast); rinse in PBS for 1 hr; dehydrate in an ethanol series; transfer to propylene oxide for 30 min; embed in resin (epon) on a glass microscope slide and place in an oven at 60º C for 48 hrs. The type specimen is deposited in the National Museum of Natural History, Smithsonian Institution, Washington, DC.

Grain size was determined on a subsample of the sediments by drying them out in a 60º C oven for 24 hrs, and then sieving them with a Gilson SS-15 sieve shaker with mesh sizes 2 mm, 1 mm, 500 µm, 250 µm, 125 µm and 63 µm. Sieve fraction weights were entered into the program GRANPLOTS with line segment (Balsille et al. 2002) and granulometric data was calculated.

## Results

### Order Macrodasyida Remane, 1925 [Rao and Clausen, 1970]. Family Lepidodasyidae Remane, 1927 [Hummon and Todaro, 2010]. Genus *Lepidodasys* Remane, 1926

#### Lepidodasys ligni sp. n.

urn:lsid:zoobank.org:act:41A72CCB-BEAD-4207-BDA5-71F36B350C05

http://species-id.net/wiki/Lepidodasys_ligni

Figs 1 - 3

##### Type locality.

Sediment from 9 m depth at Capron Shoal, Florida (27°26'52"N, 80°13'81"W), collected by Mr. Woody Lee. Granulometry as follows: Mean size 1.66 phi; SD 0.72 phi; skewness -0.95; and kurtosis 5.11. The sediments can be characterized as medium to fine grain sand with a large proportion of the sand fraction larger than the median (positive kurtosis).

##### Holotype.

Adult specimen, reproductively mature, 450 µm long; resin preparation: USNM # 1202682.

##### Paratypes.

Digital video of two adult specimens submitted to the National Museum of Natural History, Smithsonian Institution, Washington, DC. Scale patterns and reproductive anatomy visible in videos.

##### Material examined.

Eleven specimens were examined alive. Digital video was captured of three specimens. Two of the eleven specimens were prepared for phalloidin staining. One of the phalloidin-stained specimens was prepared for archival.

##### Diagnosis.

*Lepidodasys* with a strap-shaped adult body to 450 µm long. The body possesses two constrictions at approximately U06–07 and U82; the first constriction demarcates the approximate mid-region of the pharynx, and the second constriction demarcates the posterior region containing the caudal organ. Maximum body width at U06/pharyngeo-intestinal junction (PhIJ:U20)/midpoint of body (U50) is 32/44/50 µm, respectively. Pharynx to 90 µm long. Body covered with eye-shaped, non-imbricating scales arranged in a crossed-helical pattern across the dorsal and lateral surfaces; two columns of ventral interciliary scales present at midline in herringbone pattern. Up to eight anterior adhesive tubes (TbA) per side inserting directly on body surface and forming an arc toward the lateral body wall. Lateral adhesive tubes (TbL) present, up to 14 per side, beginning posterior of the PhIJ. Dorsal adhesive tubes (TbD) present, up to 11 per side, beginning at the PhIJ and extending to the caudal end. Eight posterior adhesive tubes (TbP) insert terminally on rounded caudal end. Frontal organ and large barrel-shaped caudal organ with strong circular muscles and inner canal present; ovaries and testes not observed.

##### Etymology.

The Latin *ligni* (wood) refers to Mr. William “Woody” Lee at the Smithsonian Marine Station at Fort Pierce, Florida, who assisted in the collection of the species and has been instrumental over the past decade in sublittoral collection of meiofauna for the first author (RH).

##### Description.

**Figure 1.**
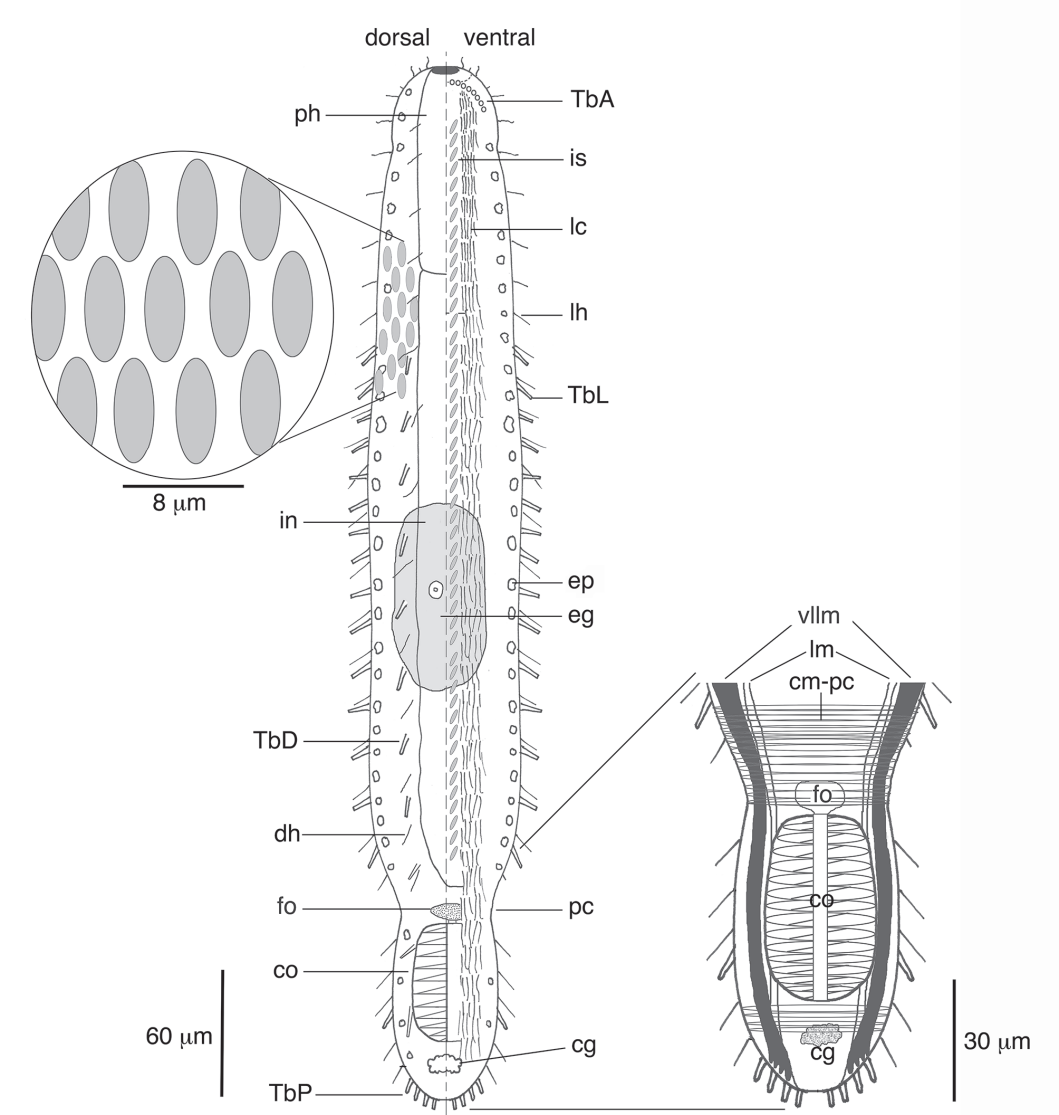
Schematic of *Lepidodasys ligni* sp. n. showing the crossed-helical scale pattern and a closeup of the musculature in the caudal region. Abbreviations: **cg** caudal gland; **cm-pc** circular musculature of the posterior constriction; **co** caudal organ; **dh** dorsal sensory hairs; **eg** mature egg; **ep** epidermal gland; **fo** frontal organ; **in** intestine; **is** interciliary scales; **lc** locomotory cilia; **lh** lateral sensory hairs; **lm** longitudinal muscle; **pc** posterior constriction; **ph** pharynx; **TbA** anterior adhesive tubes; **TbD** dorsal adhesive tubes; **TbL** lateral adhesive tubes; **TbP** posterior adhesive tubes; **vllm** ventrolateral longitudinal muscle.

**Figure 2.**
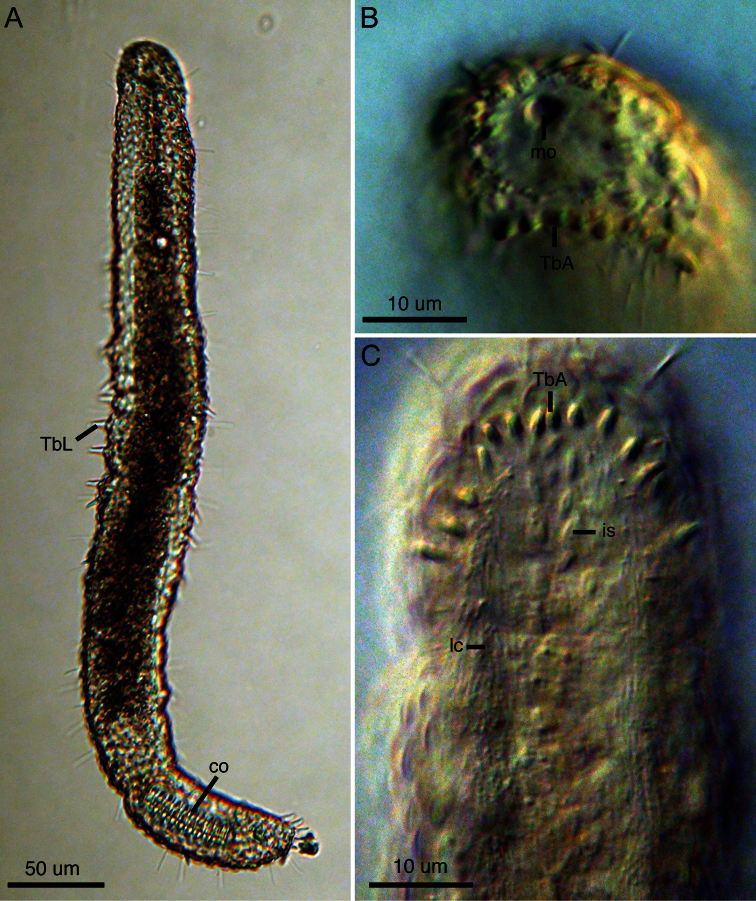
Light micrographs of *Lepidodasys ligni* sp. n. (holotype). **A** Dorsal view **B** Closeup of anterior end with differential interference contrast (DIC) **C** Ventral view of anterior end with DIC. Abbreviations: **co** caudal organ; **is** interciliary scales; **lc** locomotory cilia; **mo** mouth; **TbA** anterior adhesive tubes; **TbL** lateral adhesive tubes.

The description is based on the holotype (adult, 445 µm long; Fig. 2A), with ranges provided from specimens measured in vivo. Body strap-shaped with two notable constrictions at mid-pharynx (U06-07) and caudal end (~U82); first constriction demarcates the approximate mid-length of the pharynx and the second constriction demarcates the region of the accessory copulatory organs (Figs 1, 2A). Pharynx 90 mm long with no pharyngeal pores observed. Few sensory hairs to 8 µm long line the mouth. Stiff sensory hairs 8–15 µm are present along the ventrolateral, lateral and dorsolateral margins of the body. Ventral locomotory cilia present as two columns, each approximately 8 µm wide, that extend from ca. U05 to the posterior end (Fig. 1). Numerous small (2–5 µm) epidermal glands along the margins of the body.

**Cuticular armature.** A crossed-helical pattern of up to eight columns of elliptical scales extend across the dorsal body wall; the number of scales per column varies from 8-10 in the pharyngeal region to up to 12 in the trunk. At least three columns of elliptical scales extend around the lateral body margins and reach the ventral locomotory cilia (Fig. 1). All scales are similar in shape, lack a keel, and are approximately 7–8 µm long. None of the scales are imbricated. Two columns of ventral scales are present at the midline between the ciliary fields (Figs 1, 2C). The scales are relatively small, ca. 4–5 µm long, and oriented in a herringbone pattern in most specimens, with the anterior end of both scales farther apart from each other (ca. 5 µm) relative to their posterior ends (ca. 2–3 µm). While the herringbone pattern is evident in all specimens, some specimens had individual scales (not an entire column) oriented in a more parallel fashion (e.g., see Fig 2C); this may be evidence of individual variation in scale orientation. There was approximately 4–5 µm of space between each column and its adjacent ciliary field.

**Adhesive tubes.**Anterior adhesive tubes (TbA) distributed as a posteriorly curving arc of seven to eight tubes, 3–4 µm long, from the midline to the lateral body wall (Fig. 2C). Lateral adhesive tubes (TbL) absent from the pharyngeal region (Fig. 1). Fourteen to seventeen TbL present in the trunk region, 8–10 µm long, beginning at U28 and extending to the second body constriction. Tubes are spaced evenly down most of the trunk. A single tube is present in the demarcated region of the caudal organ, around U92. Approximately eleven evenly spaced dorsal adhesive tubes (TbD), 8–10 µm long, beginng at U28 and extending to ~U90. Eight posterior adhesive tubes (TbP), 9–10 µm long, distributed as four adhesive tubes per side on rounded caudum.

**Digestive tract.**Small terminal mouth to 5 µm wide (Fig. 2B). Pharynx to 20 µm wide and 90 µm long. Pharnygeo-intestinal junction at U20. No pharyngeal pores observed. Intestine narrow and tapering toward posterior end. Diatoms present in a single specimen. Anus at U78.

**Figure 3.**
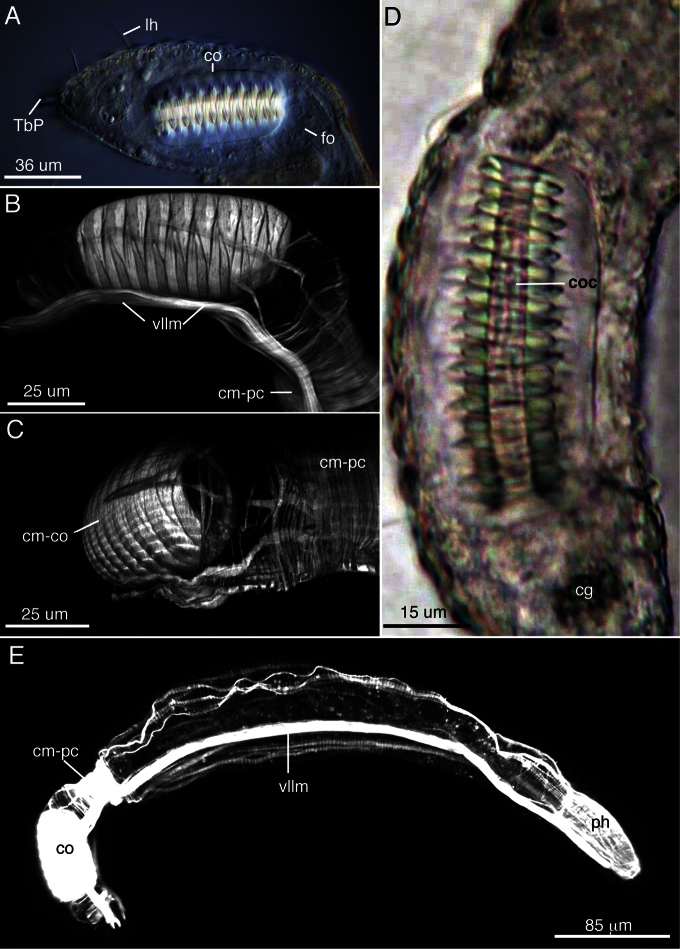
The reproductive and muscular systems of *Lepidodasys ligni* sp. n. **A** Differential interference contrast photograph of the posterior end showing the accessory reproductive organs. **B, C** Confocal images (47 × 0.35 µm optical sections) of the musculature of the posterior end in lateral (**B**) and dorsal (**C**) views **D** Closeup of the caudal organ with DIC microscopy **E** Lateral view of an entire specimen revealing the muscular system (73 × 0.4 µm optical sections). Abbreviations: **cg** caudal gland; **cm-co** circular muscles of the caudal organ; **cm-pc** circular muscles of the posterior constriction; **co** caudal organ; **coc** caudal organ canal; **dlm** dorsal longitudinal muscle; **dllm** dorsal lateral longitudinal muscle; **fo** frontal organ; **hm** helicoidal muscle (end position on midgut); **lh** lateral sensory hair; **ph** pharynx; **TbP** posterior adhesive tube; **scm** somatic circular muscles (thoughout trunk); **vllm** ventrolateral longitudinal muscle.

**Reproductive system.**Neither testes or ovaries observed. A single large egg, approximately 62 µm diameter, was present in one adult (holotype). A clear, sac-like organ interepreted as the frontal organ was present at U81–83; the organ was approximately 22 µm x 16 µm in diameter and in a single specimen appeared to contain filiform sperm. A caudal organ was present at the posterior end (U83–95), approximately 63 µm long x 30 µm wide (range: 50 µm–63 µm long, 24 µm–30 µm wide), hyaline in appearance with a straight central canal that extends along the organ’s anterior-posterior axis; the canal appears sclerotic (coc, Fig. 3D). The caudal organ is muscular. The muscle cells interdigitate to form spindle-shaped, incomplete circular fibers(Fig. 3B). A single gland, ca. 10 µm in diameter, is present posterior of the caudal organ (Fig. 3A, D).

**Muscular system.** Musculature present as circular, helicoidal and longitudinal bands. Pharynx strongly invested with splanchnic circular muscles and overlain by helicoidal bands and longitudinal muscles (Fig. 3E). The number of individual circular muscles and helicoidal bands could not be determined. In the trunk region, the helicoidal muscles extend to ca. U40 (Fig. 3E). Longitudinal muscles extend from the pharyngeal region to the caudum as three pairs of dorsal (dlm)/dorsolateral (dllm) bands (Fig. 3E). The ventrolateral longitudinal muscles (vllm) are the thickest muscles in the body and extend from ca. U03 to the caudal end (Fig. 3E). Three pairs of ventral longitudinal bands extend from the pharynx to the posterior end. All longitudinal muscles in the trunk region are surrounded by somatic circular muscles (scm) that form numerous, distinct rings down the length of the body (Fig. 3E). Beginning ca. U80-81, there is a large number (>30) of closely spaced somatic circular muscles that extend posteriorly for approximately 40 µm; the circular muscles demarcate the posterior region of the body (cm-pc) with the accessory sexual organs (Figs 1, 3B,C).

## Taxonomic remarks

The genus *Lepidodasys* consists of eight described species and several undescribed specimens from marine waters across the globe (see Hummon 2009), from as far north as the Faroe Islands (Clausen 2004) to locations along the European coastline (e.g., Remane 1926; Fregni et al. 1999; Clausen 2000; Hummon 2009) including the Baltic and Mediterranean seas (Roszczac 1939; Balsamo et al. 1994; Hummon 2009), to the Pacific waters of Japan (Lee and Chang 2011) and Hawaii (Hummon 2009), and to the Caribbean waters of Panama (Hochberg and Atherton 2011) and the Gulf of Mexico (Todaro et al. 1995).

Species of *Lepidodasys* are most easily distinguished by their slow gliding movement and their heavily sculptured cuticle, which in general consists of rounded or elliptical scales across most of their body. The shape and distribution of the scales are important taxonomic characters for distinguishing the species. Of the eight described species, six species have a crossed helical pattern (parallel pattern *sensu* Lee and Chang 2011) of scales across their dorsum: *Lepidodasys arcolepis* Clausen, 2004, *Lepidodasys castoroides* Clausen, 2004, *Lepidodasys platyurus* Remane, 1927, *Lepidodasys tsushimaenensis* Lee & Chang, 2011, *Lepidodasys worsaae* Hochberg & Atherton, 2011, and *Lepidodasys unicarenatus* Balsamo, Fregni & Tongiorgi, 1994. The species from Florida, *Lepidodasys ligni* sp. n., also bears a crossed helical pattern of scales on its dorsum. The ventral interciliary scales are in a herringbone pattern in the new species, similar to the scale arrangement in *Lepidodasys laeviacus* Lee & Chang, 2011.

Apart from scale pattern, scale shape is also an important taxonomic character, and the new species can easily be distinguished by the smooth (non-keeled) structure of its scales. The only other species with smooth scales are *Lepidodasys castoroides*, *Lepidodasys laeviacus* and *Lepidodasys tsushimaenensis*. Among these species, *Lepidodasys ligni* sp. n. is most similar to *Lepidodasys castoroides* and *Lepidodasys tsushimaenensis* in general body shape and the presence of a large caudal organ in the posterior body region. While all three species are similarly slender, *Lepidodasys tsushimaenensis* is much larger (to 730 µm) than the other two species (*Lepidodasys castoroides* to 475 µm and *Lepidodasys ligni* sp. n. to 450 µm) and lacks the notable indentation at the posterior end that demarcates the region of the accessory sexual organs. In fact, the caudal indentation gives both *Lepidodasys castoroides* and *Lepidodasys ligni* sp. n. the appearance of possessing a beaver-like tail, hence Clausen’s (2004) specific Latin epithet, *castor*. While both species possess similar “beaver tails” and caudal organs, there are several notable differences between the species: 1) the caudal organ of *Lepidodasys castoroides* possesses a cuticular endpiece that is absent in the new species; 2) there are five TbA per side in *Lepidodasys castoroides* but 7to8 TbA per side in *Lepidodasys ligni* sp. n.; 3) there are seven pairs of TbVL in *Lepidodasys castoroides* but up to 17 TbL per side (not TbVL) in the new species; and 4) there are five pairs of TbDL in *Lepidodasys castoroides* but up to eleven pairs of TbD in *Lepidodasys ligni* sp. n.

## Discussion

In addition to our taxonomic findings, this study revealed novel information on the muscular system of the new species. In contrast to previous reports (e.g., Ruppert 1978; Travis 1983), we observed somatic circular muscles in the trunk region of *Lepidodasys* (see Fig. 3E). Whether these muscles are absent from the previously examined species or were missed in examinations of their sections using transmission electron microscopy is unknown. Interestingly, the presence of a dense aggregation of somatic circular muscles in the caudal region of the new species is correlated with the position of the indentation at the posterior end, which imparts the beaver-tail like appearance. Phalloidin staining and CLSM revealed a higher number (30–40) and density (~ 1 circular muscle per 1 µm body length) of somatic circular muscles in this constricted area compared to the main trunk of the animal (11 circular muscles in 40 µm body length, ~ 0.3 circular muscles per 1 µm). It is important to state that the circular muscles in the region are likely not the cause of the constriction since this area remains delineated even when the animal is relaxed with an anaesthetic (isotonic MgCl_2_). Curiously, the muscles of this region also appear to encircle the frontal organ, and may therefore play a role in reproduction. While the identity of the frontal organ in the new species is not certain (only 1 specimen appeared to have filiform sperm within the organ), its presence near the anterior end of the caudal organ is strong correlational evidence for its identity (see also *Lepidodasys castoroides* (Clausen 2004) and *Lepidodasys tsushimaenensis* (Lee and Chang 2011)). In *Lepidodasys ligni* sp. n., the frontal organ abuts the caudal organ; the circular muscles of the caudal organ are part of the organ itself and therefore independent of the somatic circular muscles anterior to it. How these two organs function together or separately in the uptake of allosperm or release of autosperm, repsectively, is unknown and will likely require a detailed investigation with transmission electon microscopy as has been performed for other macrodasyidan gastrotrichs (e.g., Ruppert 1978, 1991; Guidi et al. 2004, 2011; Kieneke et al. 2012; Todaro et al. 2012).

## Supplementary Material

XML Treatment for Lepidodasys ligni
